# Compound small peptide of Chinese medicine alleviates cyclophosphamide induced immunosuppression in mice by Th17/Treg and jejunum intestinal flora

**DOI:** 10.3389/fmicb.2023.1039287

**Published:** 2023-03-28

**Authors:** Yuqing Cui, Lu Zhang, Ying Liu, Wei Liu, Wanyu Shi, Yongzhan Bao

**Affiliations:** ^1^College of Veterinary Medicine, Institute of Traditional Chinese Veterinary Medicine, Hebei Agricultural University, Baoding, China; ^2^Pharmacoefficacy Laboratory, Hebei Provincial Engineering Center for Chinese Veterinary Herbal Medicine, Baoding, China

**Keywords:** Chinese medicine, peptide, gut microbiota, mice, immune

## Abstract

The aim of this study was to explore the efficacy of Compound small peptide of Chinese medicine (CSPCM) on cyclophosphamide (CTX) induced immunosuppression in mice. The 100 male Kunming mice were divided into 5 groups: group A (control group), group B (model group), group C (100 mg/kg.bw CSPCM), group D (200 mg/kg.bw CSPCM) and group E (400 mg/kg.bw CSPCM). At 1–3 days, mice of group B, C, D and E were intraperitoneally injected with 80 mg/kg.bw CTX. The results showed that compared with group A, the immune organ index, body weight change, RORγ T gene expression, RORγ T protein expression, CD3^+^ cell number, Th17 number and Alpha index, white blood cell count, lymphocyte count and monocyte count were significantly decreased in group B (*p* < 0.05), while Foxp3 gene expression, Foxp3 protein expression and Treg cell number were significantly increased (*p* < 0.05), CSPCM has a good therapeutic effect on the above abnormalities caused by CTX. CTX caused the decrease of intestinal flora richness and the abnormal structure of intestinal flora, and CSPCM could change the intestinal flora destroyed by CTX to the direction of intestinal flora of healthy mice. On the whole, CSPCM has a good therapeutic effect on CTX-induced immunosuppression in mice, which is reflected in the index of immune organs, the number of T lymphocytes and Th17 cells increased, the number of Treg cells decreased and the structure of intestinal flora was reconstructed.

## 1. Introduction

CTX is one of the alkylants cytotoxic drugs including the therapeutic efficacy of cancer and autoimmune diseases (Emadi et al., 2009). It works by interfering with the immune response. However, CTX treatment is followed by immunosuppression (Yu et al., 2013; Huo et al., 2020), diabetes (Krueger et al., 2003) and alopecia (Sven Hendrix et al., 2005) side effects, whether low doses are used for prolonged periods or high doses for short periods of time (Bryniarski et al., 2009). Immune system disorder is considered the primary pathogenesis of various auto-immune diseases (Kobayashi et al., 2019). The numbers of two kinds of special CD4 + T cell subsets, T helper cell 17 (Th17) and regulator T cell (Treg) and the balance between them are important factors in maintaining the normal immune response. Th17/Treg cells are a new subset of T cells discovered recently. Th17 is a differentiated auxiliary T cell of Th 0 cells under stimulation of IL-6 and IL-23 (Huang et al., 2015). It mainly secretes pro-inflammatory factors like IL17 and IL-22, and RORγ T is an important transcriptional factor. Regulatory T lymphocytes (Treg) are an important factor for maintaining immune tolerance. They are produced by thymus and exported to the periphery, and inhibit the activation and proliferation of potential autoreactive T cells in normal bodies through active regulation, so as to regulate the body’s immunity (Cui et al., 2018). Traditional Chinese Medicine (TCM) has been used in China for millennia (Liang et al., 2020). Administration of TCM to animals can improve the animal immunity (He et al., 1992; Azzam et al., 2019), antioxidant capacity (Fu et al., 2017), intestinal flora (Luo et al., 2019; Liang et al., 2020) and effectively prevent and treat various diseases by promoting the differentiation of Th17 cells, the inhibiting differentiation of Treg cells and improving the secretion of related cytokines in the intestinal tract. Mahuang Fuzi Xixin Decoction has therapeutic effects against allergic rhinitis, possibly by regulating the intestinal microbial composition and Th17/Treg equilibrium (Liang et al., 2020). Compound sophorae decoction significantly improves the symptoms and the pathological damage of mice with colitis and influences the immune function by regulating Th17/ Treg cell balance in dextran sodium sulfate-induced colitis in mice (Xu et al., 2019). Total flavonoids of *Tetrastigma hemsleyanum* can modulate Treg/Th17 immune homeostasis to serve as an anti-inflammatory agent (Ji et al., 2019).

Maintaining the normal functioning of the intestinal barrier depends on the epithelial barrier, the intestinal immune system, the normal intestinal flora, the intestinal endocrine secretion and peristalsis (Fan et al., 2010). The most important barriers are the intestinal mucosal epithelial barrier as well as the intestinal mucosal immune barrier. Mucosal anti-infection immunity differs from the immune system, compared with the immune system, which is chronically exposed to the environment and is exposed to antigens of different origins and properties, and this requires that the mucosal immune system has the ability to discriminate between harmful and innocuous antigens, whereas for different immune responses, the mechanism and system of the immune response are also quite different (Fan et al., 2009b). The development and normal operation of intestinal immunity are inseparable from the stimulating and sustained effects of the normal intestinal flora (Omenetti and Pizarro, 2015). The differentiation and development of Th17 intestinal cells and their normal functioning are closely tied to the normal intestinal flora. Treg cells play a significant role in the gut’s immune resistance to normal flora (Xie et al., 2016). The Th17 cells and Treg cells have critical roles in the pathogenesis and disease progression of various liver diseases, respectively (Fan et al., 2009a). As a result, the equilibrium of Th17/Treg cells can be a key factor in maintaining the overall immune function of the body and the normal function of the intestinal immune barrier.

The field of Traditional Chinese Medicine (TCM) represents a vast and largely untapped resource for modern medicine (Wang et al., 2016). With improving people’s standard of living and restricting the use of antibiotics, TCM has earned unprecedented development with its benefits of safety, non-pollution, no drug resistance, and so on. The modernization of traditional Chinese medicine is developing gradually, and the CSPCM used in this experiment is a product of the modernization of traditional Chinese medicine under the guidance of traditional Chinese medicine theory. Traditional Chinese medicine (TCM) is prepared into small peptides by enzymatic hydrolysis, which not only retains the drug efficacy but also increases the rate of absorption and the rate of absorption. CSPCM is proposing a new idea for modernizing traditional Chinese medicine. In this study, CSPCM (containing 64.8% of soy peptide, 25% of wheat germ powder, 10% of Astragalus hydrolysate, and 0.2% of vitamin C) was used in mice. The manufacturing process of CSPCM used in this experiment is as follows: confirming the ratio of material to water → adding enzyme at high temperature →rotating oriented enzyme→ microfiltration→ active purification of Traditional Chinese medicine →recombination of active peptide → low temperature concentration → spray drying. The content of acid soluble protein was detected after preparation, and the acid soluble protein was required to represent over 30% of the total protein. Peptides are amino acids bound by amide bonds of compounds, to the medium of amino acids and proteins. Small peptides are compounds of two-three amino acids bound together. Some di −/tripeptides permeate through the intestinal membranes in their intact forms *via* the peptide transporter systems (Shen and Matsui, 2017). Compared with free amino acids, small peptides have the advantages of fast absorption, low energy consumption and high absorption rate (Matthews, 1975). They have an independent absorbing mechanism in the animal body and do not interfere with one another (Webb et al., 1993). The transformation of a traditional Chinese medicine into a peptide may increase its absorption rate while maintaining its potency. Traditional Chinese medicine can increase the site of action by formulating to improve its effect, but there are not many reports investigating the effects of compound peptides of Chinese medicine on the intestinal microorganisms of mice. Therefore, we studied the effect of CSPCM on intestinal flora to provide evidence for the clinical application of CSPCM.

## 2. Materials and methods

### 2.1. Chemicals

CSPCM was provided by HeBei TaiFeng Biotechnology Co., Ltd. (Handan, China). CTX CAS#6055-19-2(C_7_H_15_Cl_2_N_2_O_2_P·H_2_O) was purchased from Source Leaf Biotechnology Co., LTD(Shanghai, China). Eastep® Super Total RNA Extraction kit was purchased from Promega (Beijing) Biotechnology Co., LTD (Beijing, China). PrimeScript™ RT reagent Kit with gDNA Easer, TB Green® Premix Ex Taq™ II(Tli RNaseH Plus) were purchased from Takara Biomedical Technology (Beijing) Co., LTD (Beijing, China). BCA Protein Assay Kit was purchased from Beijing ComWin Biotechnology Co., LTD. (Beijing, China). SDS-PAGE Gel preparation kit was purchased from Biomed Genentech Inc. (Beijing, China). Rabbit Anti-Foxp3 Polyclonal Antibody, Rabbit Anti-RORγt Polyclonal Antibody and Mice Anti-GAPDH Polyclonal Antibody were purchased from Beijing Bioss Biotechnology Co. LTD (Beijing, China). FITC anti-mouse CD3 Antibody, APC anti-mouse CD4 Antibody, PE anti-mouse IL-17A Antibody, FITC anti-mouse CD4 Antibody, APC anti-mouse CD25 Antibody, PE anti-mouse FOXP3 Antibody, True-Nuclear™ Transcription Factor Buffer Set, True Nuclear TM 4X Fix Concentrate, True Nuclear TM 10X Per and True Nuclear TM Fix Diluent were purchased from BioLegend, Inc (California, United States).

### 2.2. Animals and experimental design

Male Specific Pathogen-Free (SPF) Kunming mice weighing 20.0 ± 2.0 g (6–8 w) were purchased from Spfanimals (Beijing) Laboratory Animal Science and Technology Co., LTD (Beijing, China). All mice were provided specific pathogen-free food and water *ad libitum* and acclimated for 1 week. All animal studies were approved by the Ethics Committee of Animal Experiments of Heibei Agricultural University.

After 1 week of adaptation, 100 male Kunming mice were divided into 5 groups: A (control group), group B (80 mg/ kg.bw CTX intraperitoneal injection), group C (80 mg/ kg.bw CTX intraperitoneal injection and oral 100 mg/kg.bw CSPCM), group D (80 mg/ kg.bw CTX intraperitoneal injection and oral 200 mg/kg.bw CSPCM) and group E (80 mg/ kg.bw CTX intraperitoneal injection and oral 400 mg/kg.bw CSPCM). At d1-d3, with the exception of Group A, 80 mg/kg CTX was injected intraperitoneally and Group A was injected intraperitoneally with a normal saline solution. From the fourth day of the experiment, the A group and B group oral normal saline, and the C, D and E groups were given 100 mg/kg.bw, 200 mg/kg.bw and 400 mg/kg.bw CSPCM for 14 days, respectively. After the gavage, each mouse was weighed to calculate the weight change. After the mice were sacrificed by cervical dislocation, thymus and spleen were collected and weighed, and the thymus and spleen indexes were calculated: Thymus or spleen index = thymus or spleen weight (mg)/body weight (g). The ileum was cut longitudinally and rinsed with normal saline, wrapped in foil and stored at −80°C for later use. Take mesenteric lymph nodes for flow cytometry, take ileum and intestinal contents for 16 s RNA high-throughput sequencing.

### 2.3. Western blotting analysis

The protein was extracted from the ileum tissue sample, and the protein content in the supernatant was determined by the BCA method. Then the lysate (20 μg total protein) was separated on a 10% SDS-PAGE gel and transferred to a nitrocellulose membrane. Blotting was blocked in 5% skimmed milk powder prepared in tris-buffered saline containing 0.1% Tween 20 (TBST) at room temperature for 2 h, and then with the following antibodies (anti-foxp3, anti-RORγt, anti-GAPDH) overnight at 4°C. After washing 3 times for 15 min with TBST, incubate with enzyme-labeled secondary goat anti-rabbit IgG at room temperature for 1 h. After three more 15-min washes, use the enhanced chemiluminescence detection kit to observe the immune response bands according to the manufacturer’s instructions. GAPDH served as a control. Each protein was repeated three times. Use Image J software for density measurement. The results were analyzed by the univariate analysis using IBM SPSS Statistics 19.

### 2.4. Reverse transcription quantitative polymerase chain reaction analysis

Total RNA was extracted from the ileum tissue, and cDNA was obtained by reverse transcription according to the manufacturer’s instructions. The PCR reaction was carried out using the Lightcycler®96 Real-time fluorescence quantitative instrument (Roche, Basel, Switzerland), using TB Green® Premix Ex Taq™ II. The data analysis was carried out using the 2-∆∆CT method. The housekeeping gene, β-actin, is used for normalization. The sequence of RT-qPCR primers (Takara Biomedical Technology (Beijing) Co., LTD, Beijing, China) is as follows: β-actin (5′ F: CATCCGTAAAGACCTCTATGCCAAC -3′, 5′ R: ATGGAGCCACCGATCCACA 3′);RORγt(5′F: TGACTAGGAACAGGACAGGAACC3′,5′R:CCACGGAGAGGAAAGAAGAAAA3′);Foxp3(5′F:AGTGCCTGTGTCCTCAATGGTC3′,5′R:AGGGCCAGCATAGGTGCAAG −3′). The cq value was calculated by LightCycler® 96 SW 1.1 software, and the 2-∆∆ CT value was calculated by formula. The 2-∆∆ CT value was analyzed by IBM SPSS Statistics 19 for single factor analysis.

### 2.5. Flow cytometry analysis

Pretreatment: After grinding the mesenteric lymph nodes to extract the cells, after washing with PBS, adjust the cell concentration to 1.0*106 cells/100 μl.

CD3^+^ assays: Add the FITC anti-mouse CD3 Antibody, incubate at 4°C in the dark for 30 min, add 500 μl of PBS containing 1% paraformaldehyde, and test on the machine.

Treg assays: Sample cell (1×10^6^) was resuspended by adding 100 μl of staining buffer cells were added with appropriate amounts of FITC anti-mouse CD4 antibody and APC anti-mouse CD25 antibody according to the instructions, after incubation for 30 min at room temperature in the dark, the cells were centrifuged, the supernatant was discarded, and 1 ml of 1x PBS was added to each tube to resuspend the cells again and the supernatant was discarded. Add 1 ml of ruptured membrane solution and incubate for 1 h at room temperature in the dark. After the incubation, the cells were centrifuged at 1000 rpm for 5 min, the supernatant was discarded, and the supernatant was discarded by two centrifugation with membrane disruption buffer. After resuspending the cells by adding 100 μl of breaking membrane buffer, add appropriate PE anti-mouse Foxp3 antibody according to the antibody instructions and incubate for 40–50 min at room temperature in the dark. After incubation, the supernatant was discarded by centrifugation and the cells were washed twice with membrane disruption buffer, the supernatant was discarded and 400 μl of membrane disruption buffer was added to resuspend the cells for machine detection.

Th17 assays: 0.4 μl of stimulant was added to 200 μl sample cells (1*106). Incubate at 37°C in a 5% CO_2_ incubator for 4–6 h. Mix and incubate for 30 min at room temperature in 100 μl system using 0.25 μl FITC anti-mouse CD3 antibody and APC anti-mouse CD4 antibody. Divide into two tubes, each with 100 μl of cell suspension labeled with the table antibody, numbered A1 and A2. The cells were washed by centrifugation (5 min at 1500 rpm) with the addition of 2 ml PBS and the supernatant was discarded. After resuspending the cells, add 1 ml of fixative membrane disruption solution working solution and mix again and incubate for 30 min at room temperature in the dark. The cells were washed twice by centrifugation with the addition of 2 ml of rupture buffer, and the supernatant was discarded. PE anti-mouse IL-17A antibody and isotype control IgG were added to each tube. Incubate for 30 min at room temperature in the dark. 2 ml PBS was added into each tube and centrifuged at 1500 rpm for 5 min, and the supernatant was discarded. The cells were resuspended with 0.5 ml PBS and detected on the machine.

### 2.6. Intestinal flora analysis

In this project, Illumina platform was used to conduct Epite-end sequencing of community DNA fragments, and the sequencing area was 16S_V3V4. The primer sequence is F: ACTCCTACGGGAGGCAGCA, R: GGACTACHVGGGTWTCTAA T. The resulting sequences are primed, mass filtered, denoise, spliced and de-chimed for item denoising (QIIME2). The alpha diversity index was calculated using unpumped OTU tables for 10 specified depths with 10 pumped depth values for each depth. The average score at the depth was selected as the alpha diversity index (QIIME2, R, GGplot2 package). After the ASV/OTU of each sample/group was arranged along the horizontal coordinate from large to small, the abundance value after Log2 logarithmic transformation (Log10 transformation, percentage transformation or no transformation) was used as the vertical coordinate, and the script was written in R software to draw the abundance grade curve of each sample or group. Beta diversity index is the reduction of multi-dimensional species data to one-dimensional data -- sample distance, thus representing community differences between the two samples from different perspectives (QIIME2, R, APE package). Species difference and Indicator species analysis use the abundance data of the top 20 genera in average abundance were used to calculate the clustering results of each sample and each taxon through R script, and the heat map was presented in the form of interactive graph. LEfSe analysis results included two parts, namely, the histogram of LDA value distribution of significantly different species, which was used to show the significantly enriched species (notes that the significantly down-regulated species were not shown) and their importance in each group. Cladogram, which shows the taxonomic hierarchy of designated species in each group of samples (Python LEfSe package, R language, GGtree package).

In the analysis of species composition, taxonomic statistics were based on the results of taxonomic annotations of sequence species and selected samples, and the number of taxonomic units contained in the seven taxonomic levels of phylum, class, order, family, genus and species in the species annotation results of these samples was counted. Stacked bar charts or bar charts of species composition are the most commonly used means of representing the composition of diverse species. After singleton was removed, the feature table was counted to visualize the composite distribution of each sample at the level of phylum, class, order, family, genus and species, and the analysis results were presented in histogram.

The original sequence has been submitted to NCBI. BioProject ID:PRJNA814855.

### 2.7. Statistical analysis

All of the experimental data are presented as mean ± standard deviation (SD). The results were analyzed by IBM SPSS Statistics 19 software (IBM Inc., Chicago, IL, United States) for single factor analysis. Value of *p* < 0.05 was regarded to be significantly different in statistics.

## 3. Results

### 3.1. Effects of CSPCM on immune organ indexes, body weight change and routine analysis of blood

**Figure 1:**
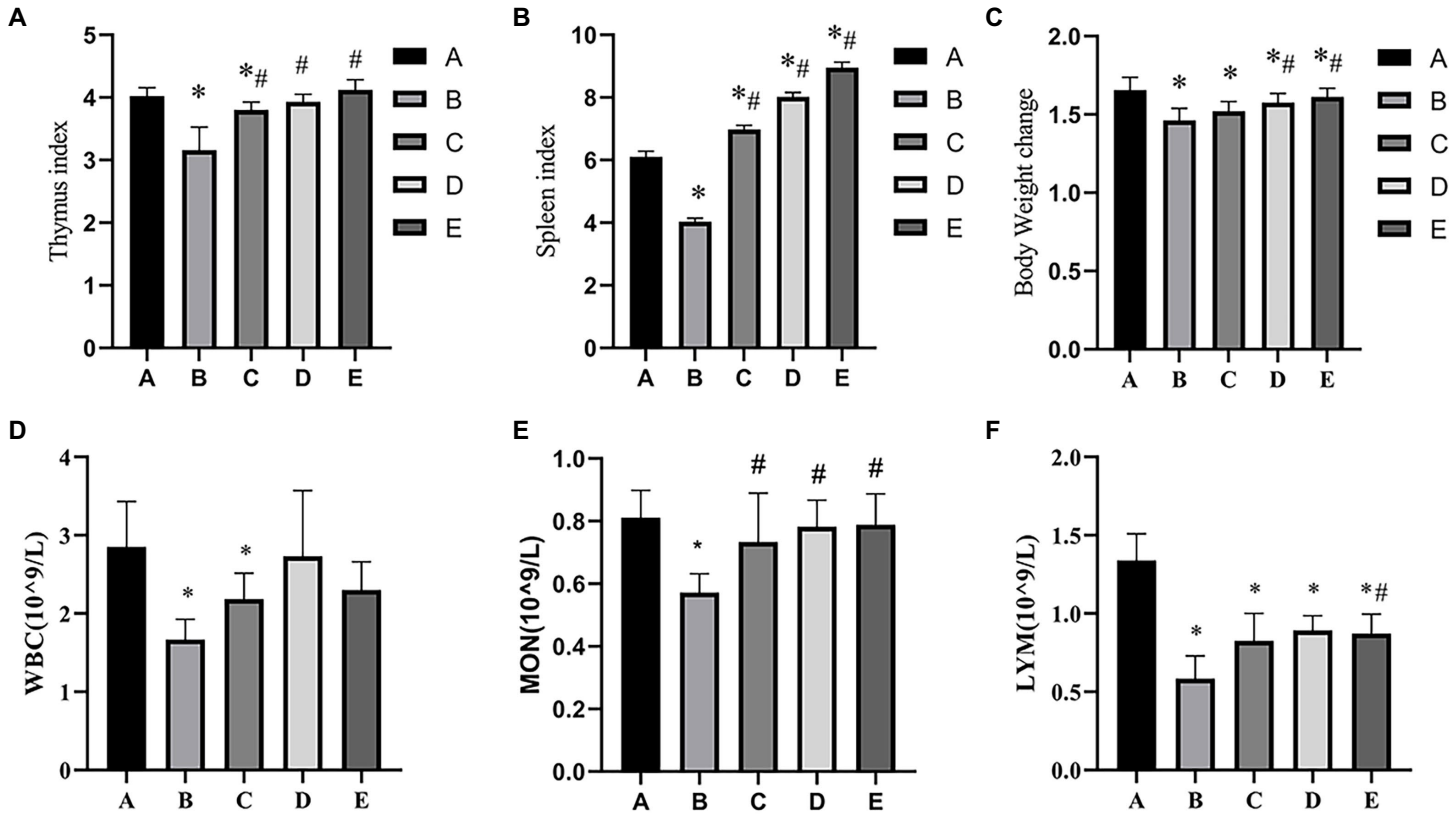
Effects of CSPCM on immune organ indexes and body weight change in mice. **(A)** Effects of CSPCM on thymus index in mice. **(B)** Effects of CSPCM on spleen index in mice. **(C)** Effects of CSPCM on body weight change on mice. **(D)** Effects of CSPCM on WBC number in mice. **(E)** Effects of CSPCM on MON number in mice. **(F)** Effects of CSPCM on the percent of LYM in mice. *: significant difference with group A (*p* < 0.05), # significant difference with group B (*p* < 0.05), same as below.

As shown in Figure 1A, the Thymus index of B and C groups was significantly lower than that in the A group (*p* < 0.05), the Thymus index of D and E groups were significantly higher than that in the B group (*p* < 0.05). As shown in Figure 1B, the Spleen index of B group was significantly lower than that in the A group (*p* < 0.05), the Spleen index of C, D and E groups was significantly higher than that in A and B groups (*p* < 0.05). As shown in Figure 1C, the body weight change of B and C groups was significantly lower than that in the A group (*p* < 0.05), the body weight change of D and E groups were significantly higher than that in the A and B groups (*p* < 0.05). As shown in Figures 1D–F),the number of WBC, MON and LYM in B group was significantly lower than that in the A groups (*p* < 0.05), the number of WBC in C group was significantly higher than that in the B groups (*p* < 0.05), the number of WBC and LYM in C, D and E groups was significantly higher than that in the B groups (*p* < 0.05).

### 3.2. Effects of CSPCM on relative genes and proteins expression

**Figure 2:**
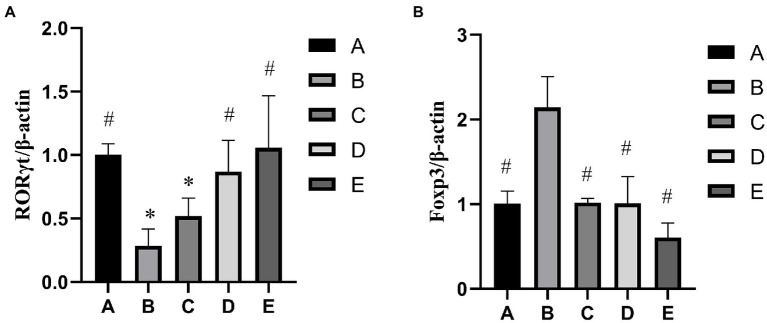
Effects of CSPCM on relative genes expression. **(A)** Effects of CSPCM on RORγt/β-actin gene expression in mice. **(B)** Effects of CSPCM on Foxp3/β-actin gene expression in mice.

As shown in Figure 2A, the RORγt gene expression of B and C groups was significantly lower than that in the A group (*p* < 0.05), the RORγt gene expression of D and E groups were significantly higher than that in the B group (*p* < 0.05). As shown in Figure 2B, the Foxp3 gene expression of A, C, D and E groups was significantly lower than that in the B group (*p* < 0.05).

**Figure 3:**
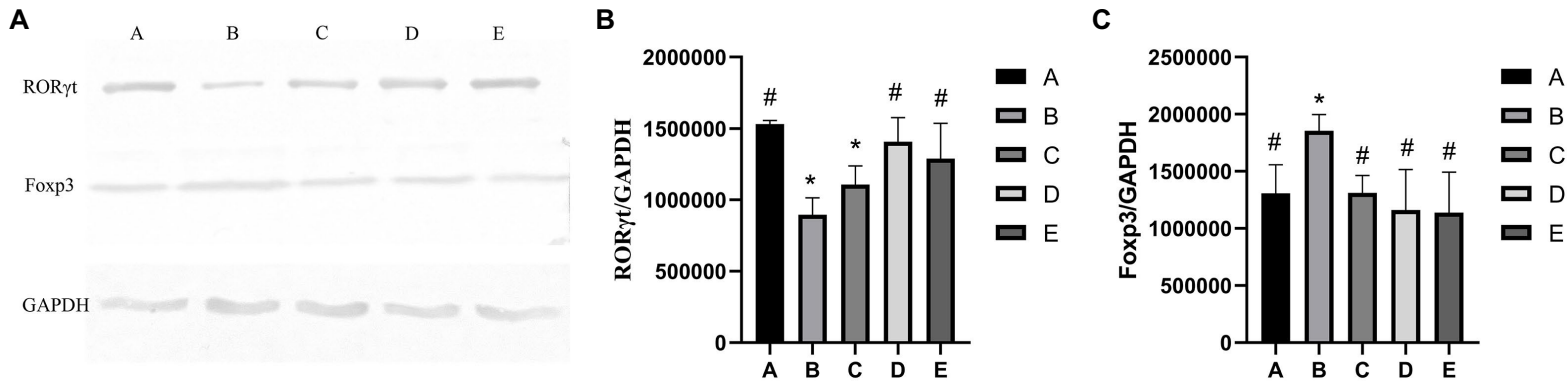
Effects of CSPCM on relative proteins expression. **(A)** Western blot analysis of RORγt, Foxp3 and GAPDH **(B)** Effects of CSPCM on RORγt/GAPDH protein expression in mice. **(C)** Effects of CSPCM on Foxp3/GAPDH protein expression in mice.

As shown in Figure 3B, the RORγt protein expression of B and C groups was significantly lower than that in the A group (*p* < 0.05), the protein gene expression of D and E groups were significantly higher than that in the B group (*p* < 0.05). As shown in Figure 3C, the Foxp3 gene expression of A, C,D and E groups was significantly lower than that in the B group (*p* < 0.05), the Foxp3 gene expression of B group was significantly higher than that in the A group (*p* < 0.05).

### 3.3. Effects of CSPCM on expression of surface antigen in mesenteric lymph node cells

#### 3.3.1. CD3^+^

**Figure 4:**
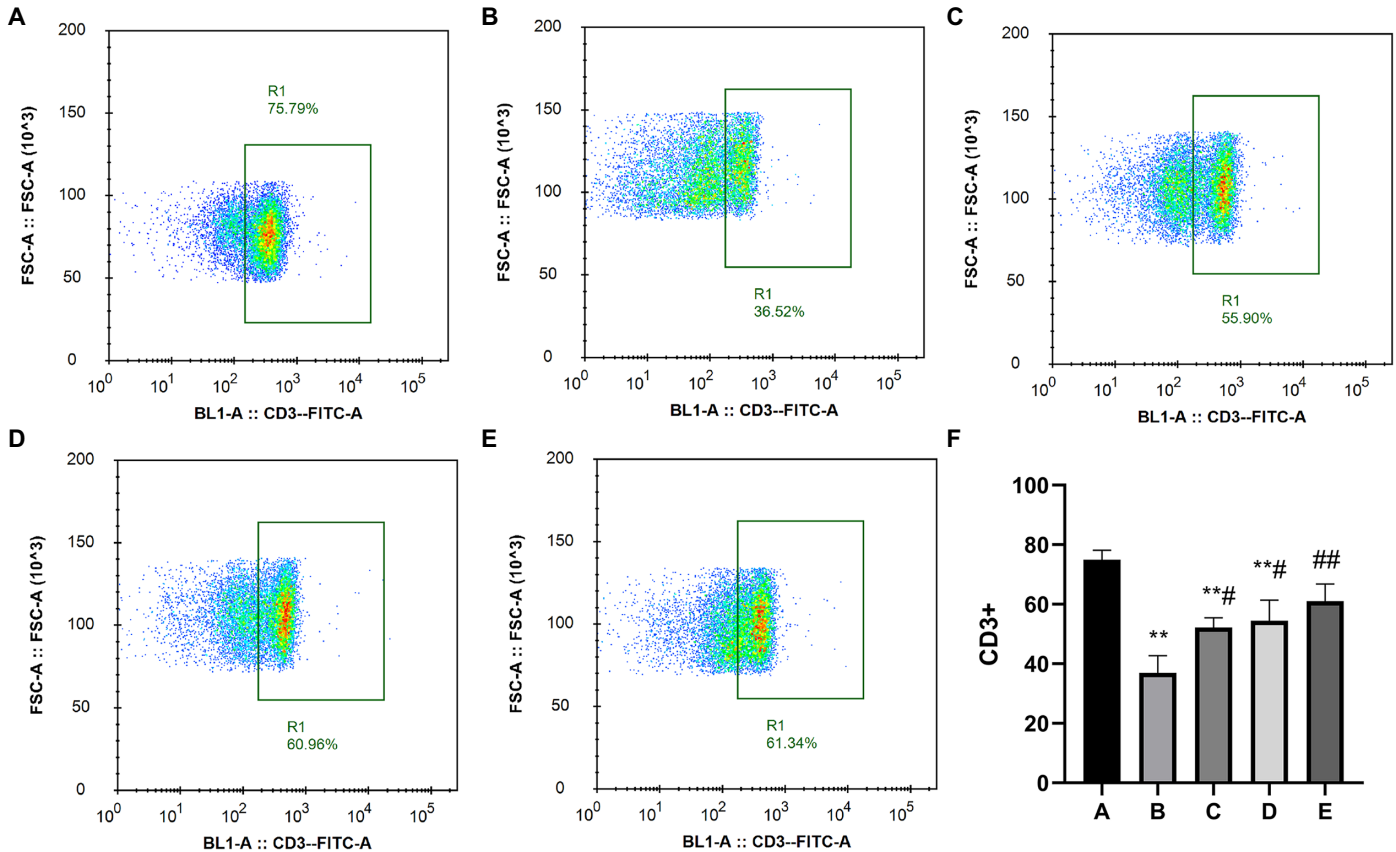
Effects of CSPCM on the percentage of CD3^+^ cells in Mesenteric lymph nodes. **(A)** The percentage of CD3+ cells in group A. **(B)** The percentage of CD^3+^ cells in group B. **(C)** The percentage of CD3+ cells in group C. **(D)** The percentage of CD3+ cells in group D. **(E)** The percentage of CD3+ cells in group E. **(F)** Effect of CSPCM on percentage of CD^3+^ cells in each group.

As shown in Figure 4, the percentage of CD^3+^ cells in the A, C, D and E groups was significantly higher than that in the B group (*p* < 0.05).

#### 3.3.2. Th17

**Figure 5:**
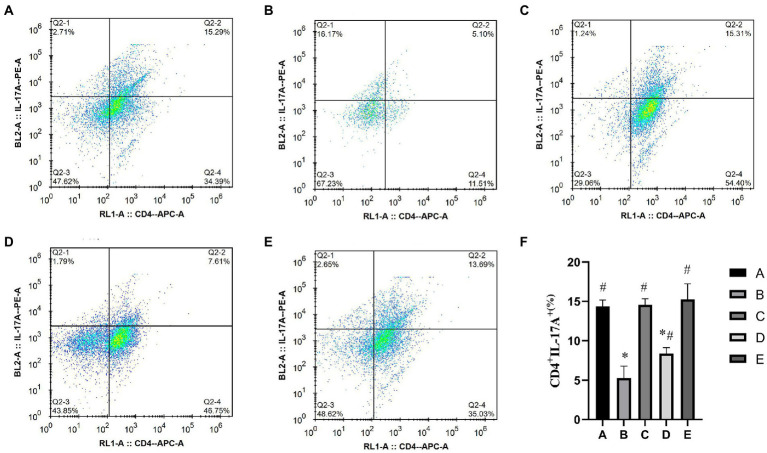
Effects of CSPCM on the percentage of Th17 cells in Mesenteric lymph nodes. **(A)** The percentage of Th17 cells in group A. **(B)** The percentage of Th17 cells in group B. **(C)** The percentage of Th17 cells in group C. **(D)** The percentage of Th17 cells in group D. **(E)** The percentage of Th17 cells in group E. **(F)** Effect of CSPCM on percentage of Th17 cells in each group.

As shown in Figure 5, the percentage of CD4^+^ IL17^+^ Th17 cells in the A, C, D and E groups was significantly higher than that in the B group (*p* < 0.01), the percentage of CD4^+^ IL17^+^ Th17 cells in the A and C groups was significantly lower than that in the A group (*p* < 0.05).

#### 3.3.3. Treg

**Figure 6:**
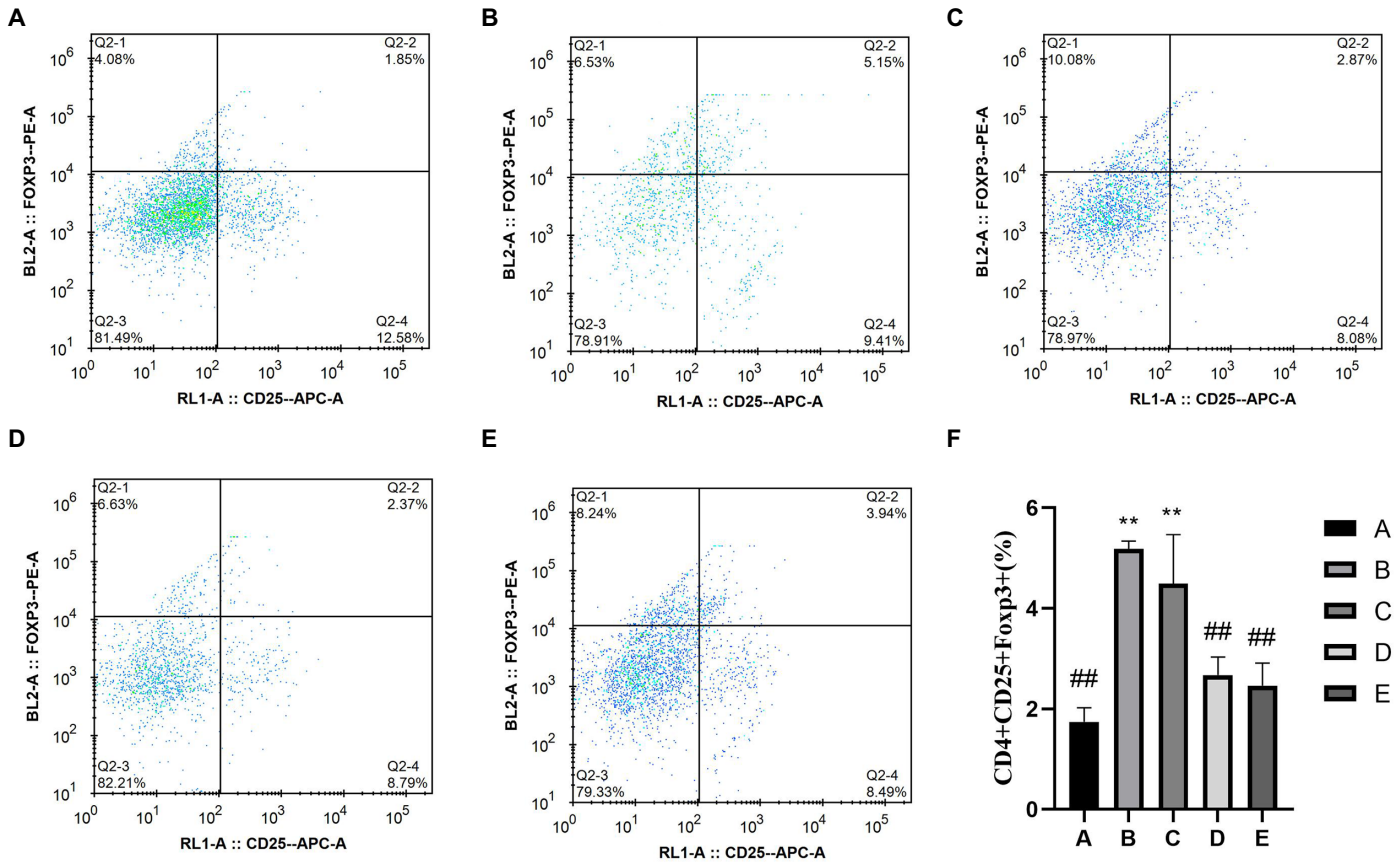
Effects of CSPCM on the percentage of Treg cells in Mesenteric lymph nodes. **(A)** The percentage of Treg cells in group A. **(B)** The percentage of Treg cells in group B. **(C)** The percentage of Treg cells in group C. **(D)** The percentage of Treg cells in group D. **(E)** The percentage of Treg cells in group E. **(F)** Effect of CSPCM on percentage of Treg cells in each group.

**Figure 7:**
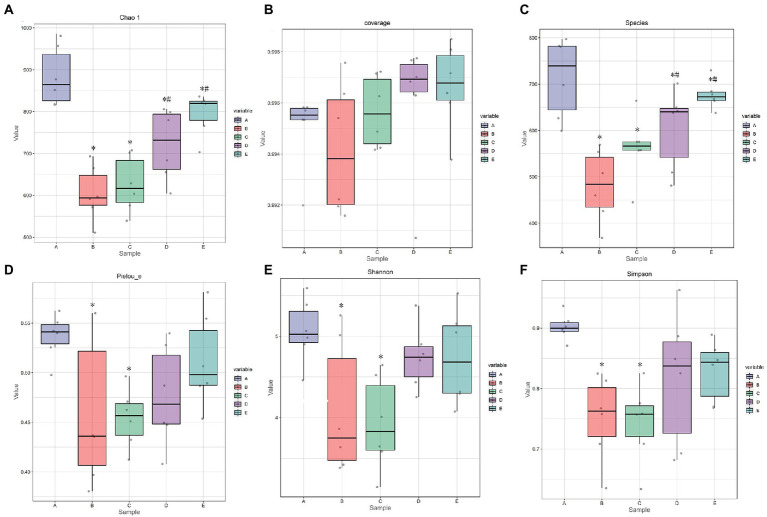
The effect of CSPCM on Alpha index in mice samples. **(A)** The effect of CSPCM on Chao1 index. **(B)** The effect of CSPCM on Coverage index. **(C)** The effect of CSPCM on Species. **(D)** The effect of CSPCM on Pelou-e index. **(E)** The effect of CSPCM on Shannon index. **(F)** The effect of CSPCM on Simpson index.

The detection of CD4^+^CD25^+^Foxp3treg cells was divided into two steps: First, CD4^+^ cells were screened (Figure 6), and then CD25^+^Foxp3Treg cells were screened (Figure 7). The percentage of CD4^+^CD25^+^Foxp3^+^Treg cells in the D and E groups was significantly lower than that in the B group (*p* < 0.05), the percentage of CD4^+^CD25^+^Foxp3^+^Treg cells in the B and C groups was significantly higher than that in the A group (*p* < 0.05).

### 3.4. Effects of CSPCM on gut microbiota

#### 3.4.1. Alpha diversity analysis

As shown in Figures 6A,C, the chao1 index and observed species of B, C, D and E groups were significantly lower than that in the A group (*p* < 0.05), the chao1 index and observed species of D and E groups were significantly higher than that in the B group (*p* < 0.05). As shown in Figure 6D, the Pielou-e index B and C groups were significantly lower than that in the A group (*p* < 0.05). As shown in Figures 6E,F, the Shannon index and Simpson index of B, C, D and E groups were significantly lower than that in the A group (*p* < 0.05).

On the whole, rank abundance is gentle, indicating high evenness of community composition (Figure 7A). As shown in Figure 7B, the number of common OTUs is 290. The number of OTU specific to group A, B, C, D and E were 1911, 907, 1,451, 1,025, and 811, respectively.

#### 3.4.2. Beta diversity analysis

**Figure 8:**
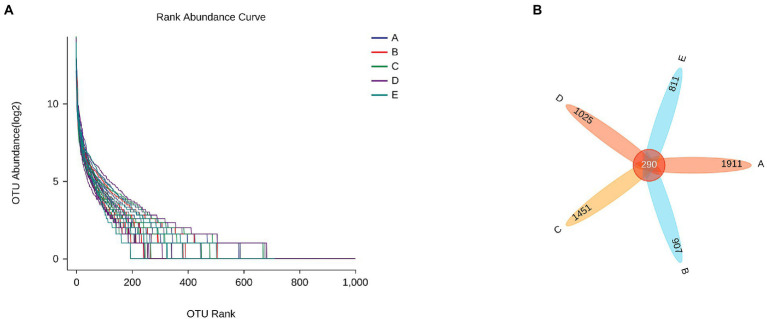
The effect of CSPCM on Rank abundance and OTU numbers in mice samples. **(A)** The effect of CSPCM on Rank abundance. **(B)** The effect of CSPCM on OTU numbers.

Beta diversity uses Principal coordinate analysis (PCoA) and Nonmetric Multidimensional scaling (NMDS) to reduce the dimensionality of multi-dimensional microbial data and show the main trend of data changes. Figure 8 shows that intraperitoneal injection of cyclophosphamide in mice can significantly change the intestinal flora relative to group A. After CSPCM is used, the intestinal flora of mice gradually becomes normal.

#### 3.4.3. Species differences and marker species analysis

**Figure 9:**
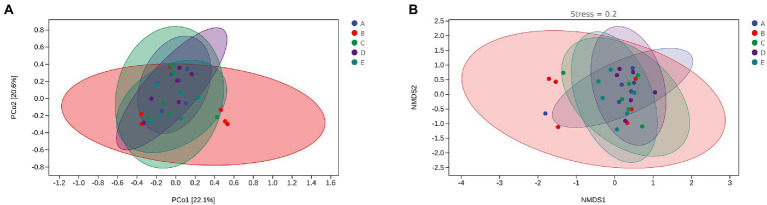
The effect of CSPCM on Beta diversity in mice samples. **(A)** PCoA analysis of mice intestinal microflora. **(B)** NMDS analysis of mice intestinal microflora.

As shown in Figure 9A, *Allobaculum*, *Bifidobacterium*, *Oscillospira*, *Roseburn*, *Adlercreutzia*, and *Desulfovibrio* were abundant in group A. *Allobaculum*, *Bifidobacterium*, *Oscillospira*, *Lactobacillus*, *Prevotella* and *Faecalibacterium* were abundant in group B. *Streptococcus*, *Corynebacterium*, *Burkholderia*, *Aquabacterium*, *Coprobacillus*, *Rubrivivax* and *Elsera* were abundant in group C. [*Ruminococcus*], *Coprococcus*, *Streptococcus*, *Corynebacterium*, *Burkholderia* and *Aquabacterium* were abundant in group D. *Faecalibacterium*, *Candidatus arthromitus* and *Bacteroides* were abundant in group E.

**Figure 10:**
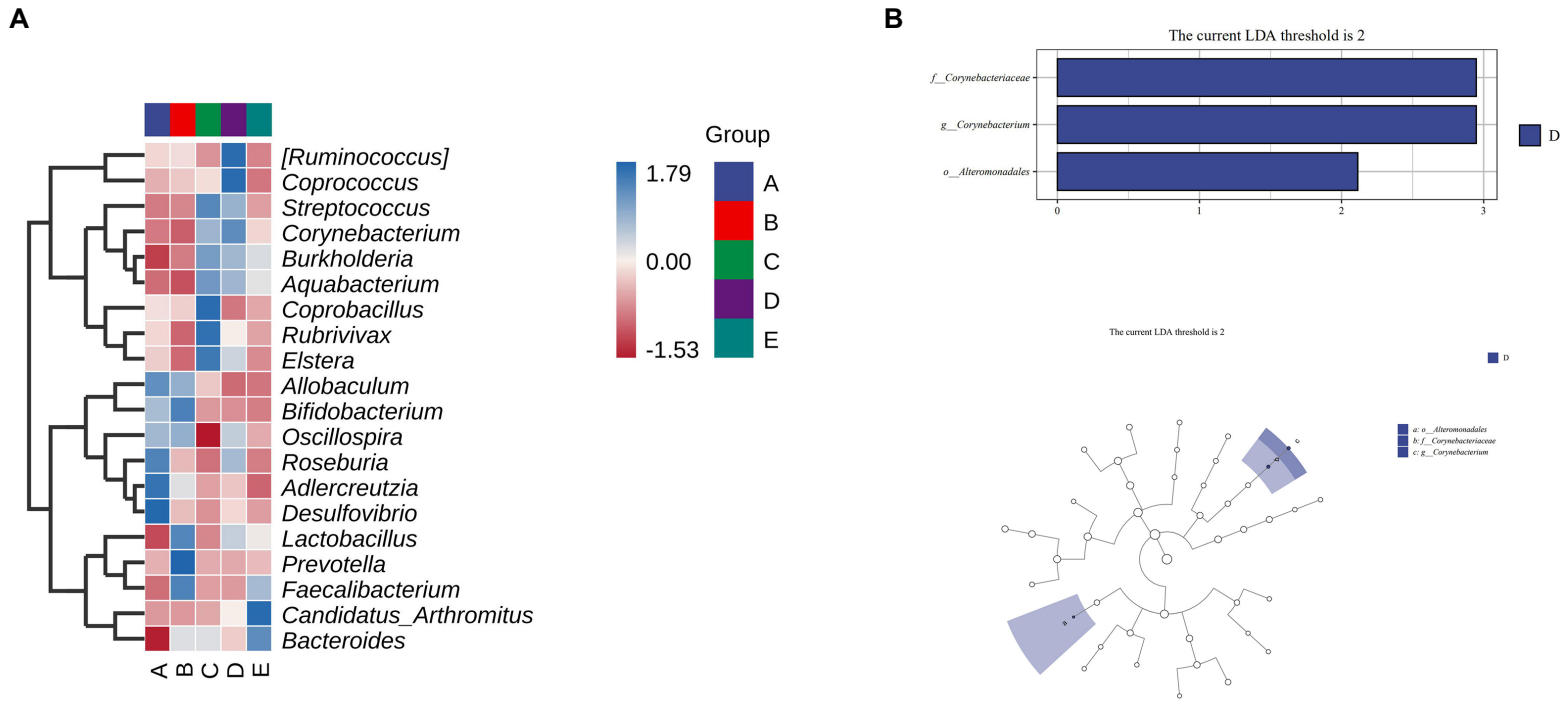
The effect of CSPCM on Species differences and marker species analysis in mice samples. **(A)** Heat map of mice intestinal microflora. **(B,C)** LEfSe analysis of intestinal flora.

To determine the significant increased bacteria in group A or others, supervised comparisons by LEfSe (LDA > 2.0) were performed. In Figure 9, *Corynebacteriaceae* (at family level), *Corynebacterium* (at genus level) and *Alteromonadales* (at order level) in group D were significantly higher than that in other groups (Figure 10).

#### 3.4.4. Analysis of taxonomic composition

**Figure 11:**
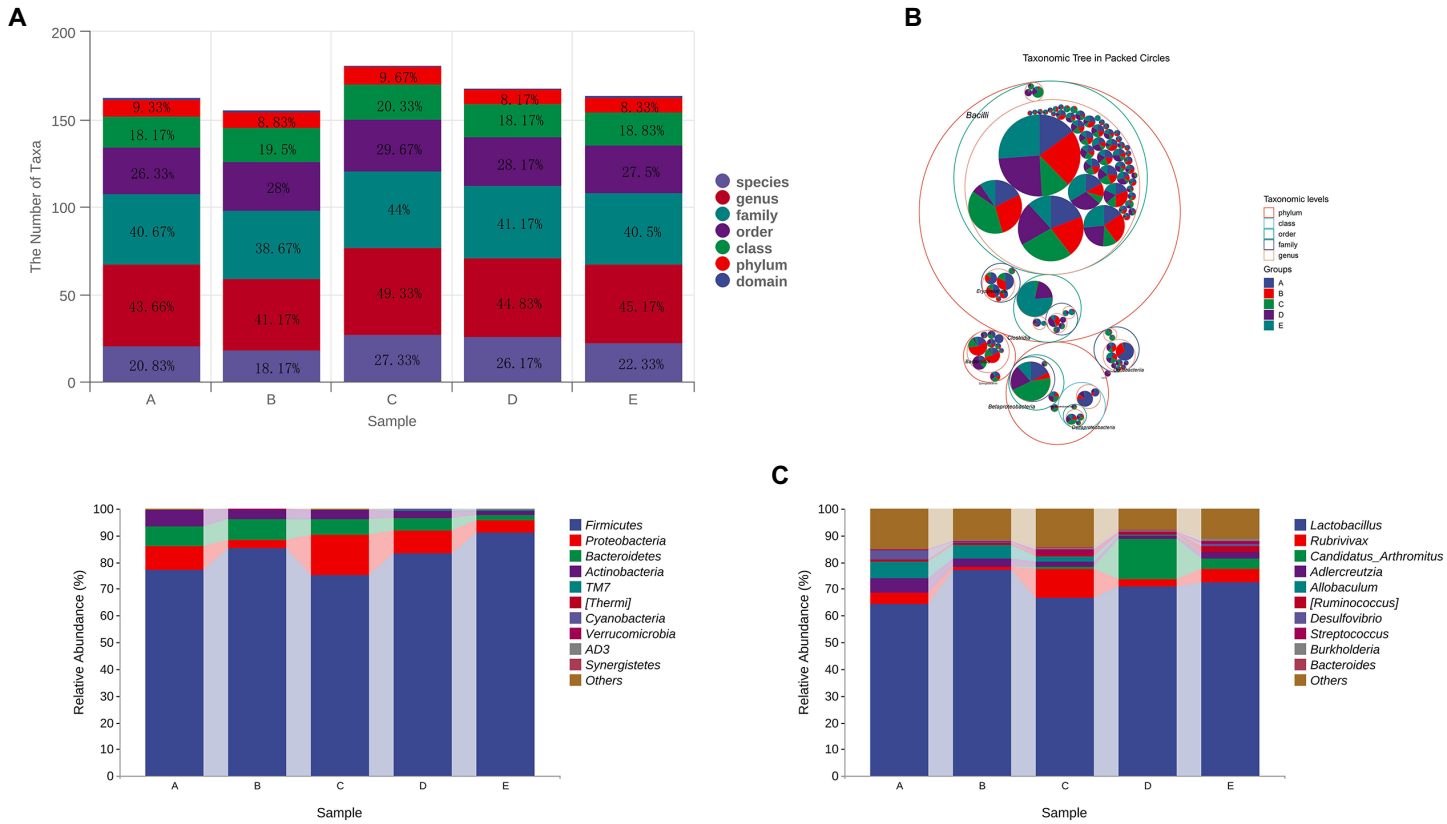
The effect of CSPCM on taxonomic composition in mice samples. **(A,B)** The effect of CSPCM on taxonomic composition in mice samples. **(C)** Effect of CSPCM on intestinal flora composition at phylum level. **(D)** Effect of CSPCM on intestinal flora composition at genus level.

Compared with group A, the number of taxa in group B decreased by 1, 2, 2.49 and 2.66% at phylum, family, genus and species levels, respectively. After treatment with different doses of CSPCM, the number of taxa in group C increased by 0.84, 0.83, 1.67, 5.33, 8.16 and 9.16% at phylum, class, order, family, genus and species levels, respectively, compared with group B. Compared with group B, the number of taxa in group D increased by 17,2.5,3.66 and 8% at order, family, genus and species levels, respectively. Compared with group B, the number of taxa in group E increased by 1.83, 4 and 4.16% at family, genus and species level, respectively (Figures 11A,B).

At the phylum level, the main bacteria in each group were *Firmicutes*, *Proteobacteria*, *Bacteroidetes* and *Actinobacteria*. The abundance of these four bacteria in groups A,B,C,D and E was 99.42, 98.98,99.6,99.33 and 99.07%, respectively (Figure 11C).

At the genus level, the top 5 genera in abundance was *Lactobacillus*, *Rubrivivax*, *Candidatus Arthromitus*, *Adlercreutzia* and *Allobaculum*. Compared with group A, the abundance of *Lactobacillus* in Group B increased by 12.62%, while *Rubrivivax*, *Adlercreutzia* and *Allobaculum* decreased by 3.05, 2.24 and 1.14%, respectively. Compared with group B, the abundance of *Lactobacillus*, *Adlercreutzia* and *Allobaculum* in group C decreased by 10.48, 1.23 and 2.97%, *Rubrivivax* and *Candidatus Arthromitus* increased 9.9 and 0.71%, respectively. Compared with group B, the abundance of *Lactobacillus*, *Adlercreutzia* and *Allobaculum* in group D decreased by 4.62, 0.82 and 4.75%. *Rubrivivax* and *Candidatus Arthromitus* increased 3.8 and 4.04%, respectively. Compared with group B, the abundance of *Lactobacillus*, *Adlercreutzia* and *Allobaculum* in Group E decreased by 6.36, 1.9 and 4.55%. *Rubrivivax* and *Candidatus Arthromitus* increased by 1.65 and 15.18%, respectively (Figure 11D).

## 4. Discussion

CTX is an anti-tumor drug to treat malignant tumors, as well as an immunosuppressive agent to treat various auto-immune diseases (Chen et al., 2020). CTX is the first wide-spectrum “latent” antitumor medication. It is inactive *in vitro* and works by the liver after penetrating the animal body (Ding et al., 2019). The therapeutic effect of CTX is important, but its side effects are devastating as well. After CTX treatment, emaciation and hair loss can be seen intuitively (Xie et al., 2016; Shi et al., 2017), and invisible changes in the body including the immune system and intestinal flora have also been disrupted (Ying et al., 2020). Traditional Chinese Medicine (TCM) has been developed in China for thousands of years, and its mechanism of action is less clear than modern medicine. As a practice medicine, although the mechanism of TCM is not clear, its role in treating diseases is beyond doubt (Jianxin et al., 2011; Yu et al., 2018; Che et al., 2019; Guo et al., 2019).

Long-term intraperitoneal injection and short-term high-dose intraperitoneal cyclophosphamide injection have been reported to produce immune-suppressive mouse models (Ding et al., 2019). The results showed that compared with the control group, the immune organ index and body weight changes in the model group were significantly reduced, and the CD3+ cells in the mesenteric lymph nodes were significantly reduced. Immune organ indexes most intuitively reflect the body’s immune level. The CD3+ is a specific T cell antibody that is expressed on the surface of all T cells (Jamwal et al., 2020). The blood routine demonstrated that cyclophosphamide significantly decreased white blood cells, lymphocytes and monocytes. Therefore, the proportion of CD3+ cells directly reflects the immune level of the body, indicating the success of the immune suppressant model preparation in this experiment.

The therapeutic effect of CSPCM is reflected in the increase in the index of immune organs. Thymus is the organ that produces T cells, and spleen is the largest lymphoid organ in the body, rich in T cells and B cells, so at the same time, CSPCM increases the number of CD3^+^ cells. The 100 mg/kg, 200 mg/kg and 400 mg/kg CSPCM all increased the number of leukocytes, lymphocytes, and monocytes in the blood to varying degrees.

Th17 cells are characterized by the expression of the transcription factor retinoic acid-related orphan receptor (RORγt) and Treg cells are characterized by the expression of the forkhead box O3a (Foxp3). Results of RT-PCR, Western blot and flow cytometry showed that CTX caused dysregulation of the Th17/Treg ratio, and CSPCM increased the number of Th17 cells and decreased the number of Treg cells, remodeling the balance of Th17/Treg ratio *in vivo*. The intricate balance established between Th17 cells and Treg cells, which is critical for maintaining immune homeostasis, has recently attracted increasing attention in regulating metabolic disorders (Blacher et al., 2017; Hall et al., 2017). Treg cells were generated from the thymus and exported to the periphery, inhibiting the activation and proliferation of potential self-reactive T cells existing in the normal body through active regulation. The 17 cells play an essential role in the host’s defensive responses. There are different mechanisms of action for TCM and antibiotics. Antibiotics restore health by directly inhibiting harmful bacteria. The target of TCM is the animal body, which may regulate the animal body and mobilize the immune system or other systems to withstand disease. TCM theory is guided by the Centripetal vector/centrifugal vector and its derivative dynamics(C/C.D.) and five kinds of steady-state space dynamics induced by centripetal vector and centrifugal vector (F.S.C.), which pays attention to the cause of disease in the body is “imbalance “, whether it is the imbalance among the functions of various organs and even cells in the animal body or the imbalance between the animal body and the environment, which will cause the occurrence of disease. For example, a dysregulated relationship Th17/ Treg cellular *in vivo* contributes to the progression of the disease. And the role of traditional Chinese medicine is to help the body re-establish balance and withstand illness.

Based on the results of the gut flora, the effects of CTX are also extremely destructive in terms of modifications of the gut flora. From the alpha index, CTX reduces not only the diversity of the gut flora, but also the richness of the species. Moreover, the number of OTUs, the level of phylum, the level of the family, the level of the genus and the level of the intestinal flora of the species have decreased. For a comprehensive evaluation of microbial community diversity, the Chao1 index and the observed species index were used to represent richness, the Shannon index and the Simpson index were used to represent diversity. The Pielou Evenness Index was used to represent uniformity, while the Good Coverage Index was used to represent coverage. The algorithm adopted in this experiment is that the higher the score, the higher the richness, diversity, consistency and coverage. The results showed that species richness, diversity, evenness and coverage increased linearly with the addition of CSPCM, and CSPCM improved the coverage even more than control group.

Analysis of intestinal flora composition results were consistent with alpha scores, and species taxa decreased after intraperitoneal injection of CTX and improved after CSPCM treatment. Species compositional analysis determines the difference between taxa and phylum and genus. After intraperitoneal injection of CTX, the abundance of *Lactobacillus*, as a beneficial bacterium, increased, and the number of *Lactobacillus* decreased after CSPCM treatment. However, study showing that having a higher proportion of *Lactobacillus* alone does not guarantee greater physical health, but having a stable intestinal flora seems to be important for health (Cheng et al., 2019). When there is too much *Lactobacillus*, it will also become pathogenic bacteria. *Lactobacillus* decomposes sugars to produce acids and inhibits the growth of other bacteria, which may account for the decrease of group B species and *proteobacteria*. Excess *Lactobacillus* may disturb the balance of bacteria in the intestine, leading to poor digestion, electrolyte balance and even an increased risk of diarrhea. Studies have shown that short-chain fatty acids can contribute to Treg cell production, resulting in an imbalance in the Th17/Treg cell ratio (Cheng et al., 2019). However, the application of CSPCM can adjust the abundance of lactic acid bacteria to the normal level, reshape the balance of Th17/ Treg cell ratio, and achieve the effect of improving immunosuppression. The intestine is a complex ecosystem composed of intestinal epithelium, immune cells, mucous layer and microbial communities (Jiao et al., 2020). Recent studies have identified gut flora as an important factor in boosting the immune system (Kishida et al., 2018). The *Candiatus-Arthromitus* is a kind of segmented filamentous bacteria are tightly anchored to epithelial cells of the ileal mucosa and possess the unique ability to specifically modulate the host immune response. Segmented filamentous bacteria activate various immune functions, leading to innate immune responses (Keilbaugh and Banchereau, 2005; Gaboriau-Routhiau et al., 2009), increasing and activating cytotoxicintraepithelial lymphocyte populations (Umesaki et al., 2013), and coordinating T-cell responses, including the specific induction of Th17 cells (Thompson et al., 2013). Improvement of the immune function of mesenteric lymph nodes in groups D and E can be achieved by increasing the abundance of *Candiatus*-Arthromitus in the gut.

## 5. Conclusion

CSPCM has a good therapeutic effect on CTX-induced immunosuppression in mice, which is reflected in many aspects: the index of immune organs, the number of T lymphocytes and Th17 cells increased, the number of Treg cells decreased and the structure of intestinal flora was reconstructed. CSPCM has great research and development potential as a treatment for CTX-related immunosuppression.

## Data availability statement

The datasets presented in this study can be found in online repositories. The names of the repository/repositories and accession number(s) can be found below: https://www.ncbi.nlm.nih.gov/genbank/, PRJNA814855.

## Ethics statement

The animal study was reviewed and approved by Ethics Committee of Animal Experiments of Heibei Agricultural University.

## Author contributions

YC: conceptualization ideas, methodology, and writing–original draft. LZ: software, validation, and investigation. Chunyu Lu: data curation. WL: data curation. YB and WS: project administration and funding acquisition. Zhanjun Wu: visualization. All authors contributed to the article and approved the submitted version.

## Funding

The work was supported by Graduate Student innovation ability Training funded project of Hebei Education Department-- Effects of Compound small peptide of Chinese medicine on intestinal immunity and intestinal microflora in mice (CXZZBS2022044).

## Conflict of interest

The authors declare that the research was conducted in the absence of any commercial or financial relationships that could be construed as a potential conflict of interest.

## Publisher’s note

All claims expressed in this article are solely those of the authors and do not necessarily represent those of their affiliated organizations, or those of the publisher, the editors and the reviewers. Any product that may be evaluated in this article, or claim that may be made by its manufacturer, is not guaranteed or endorsed by the publisher.
