# Supplementary figures and images for: Compound small peptide of Chinese medicine alleviates cyclophosphamide induced immunosuppression in mice by Th17/Treg and jejunum intestinal flora
Source: Front Microbiol. 2023 Mar 28;14:1039287. doi: 10.3389/fmicb.2023.1039287 (PMC10089124; doi:10.3389/fmicb.2023.1039287)

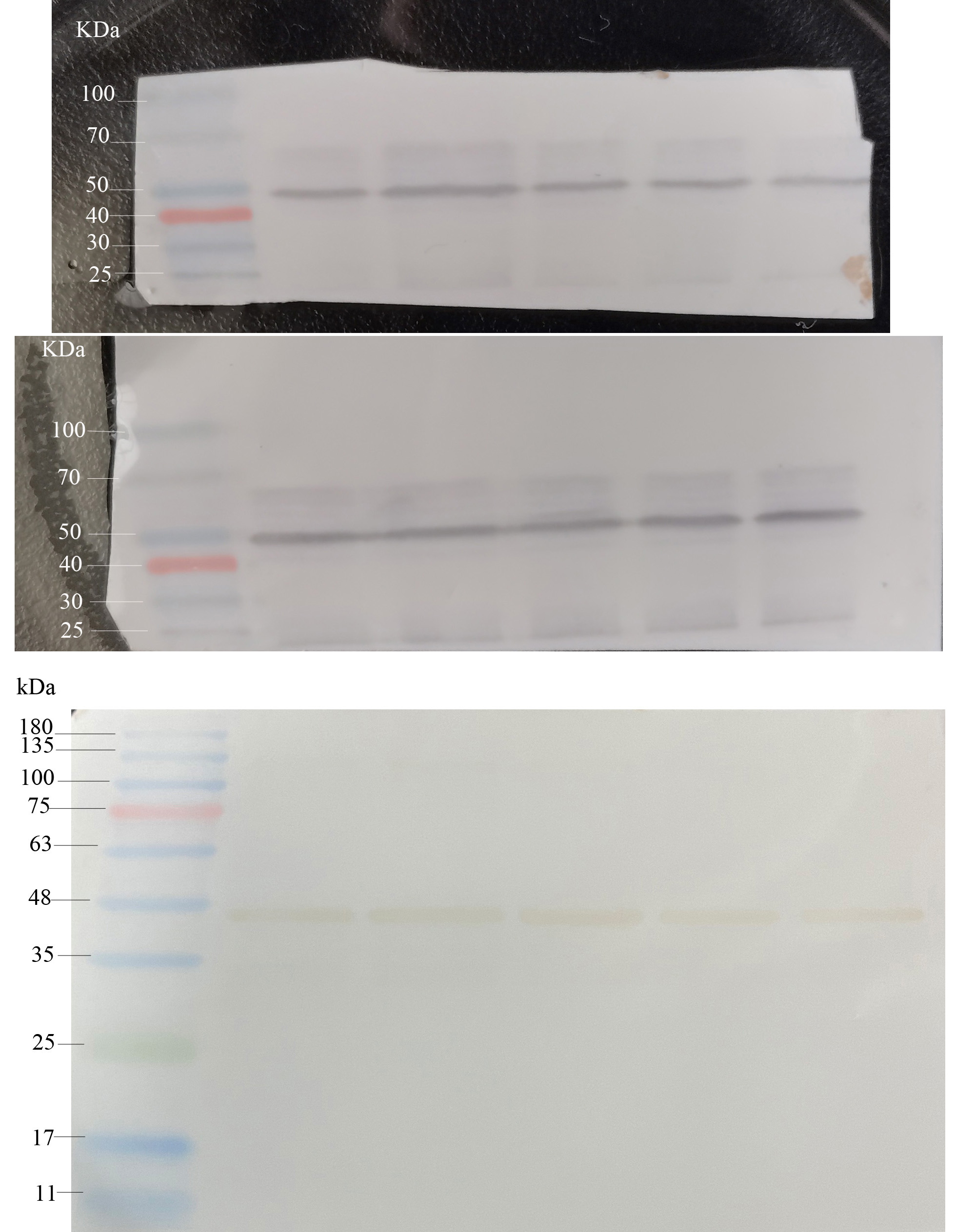

Supplement: Supplementary file 1 [file Image_1.JPEG]

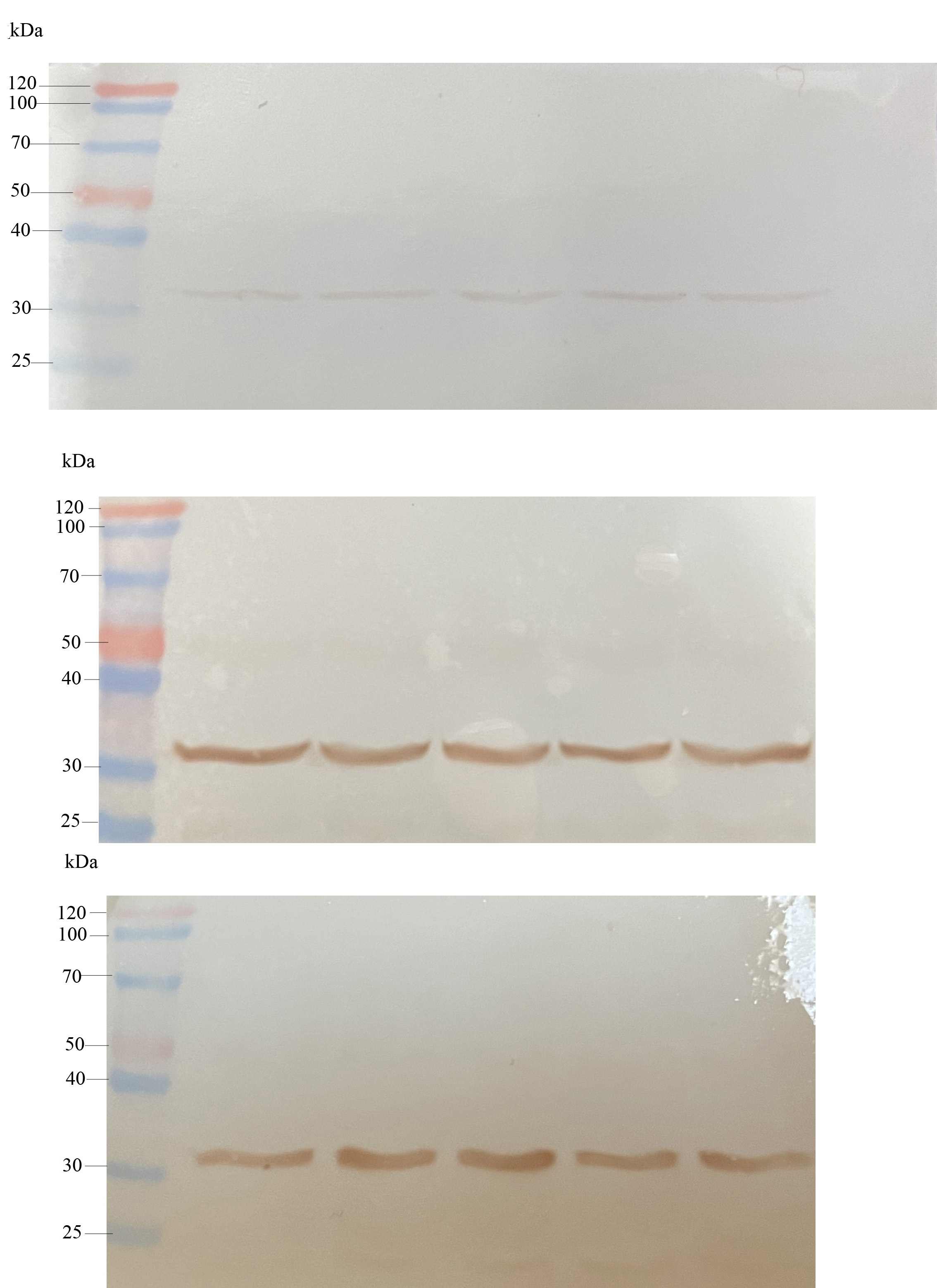

Supplement: Supplementary file 2 [file Image_2.JPEG]

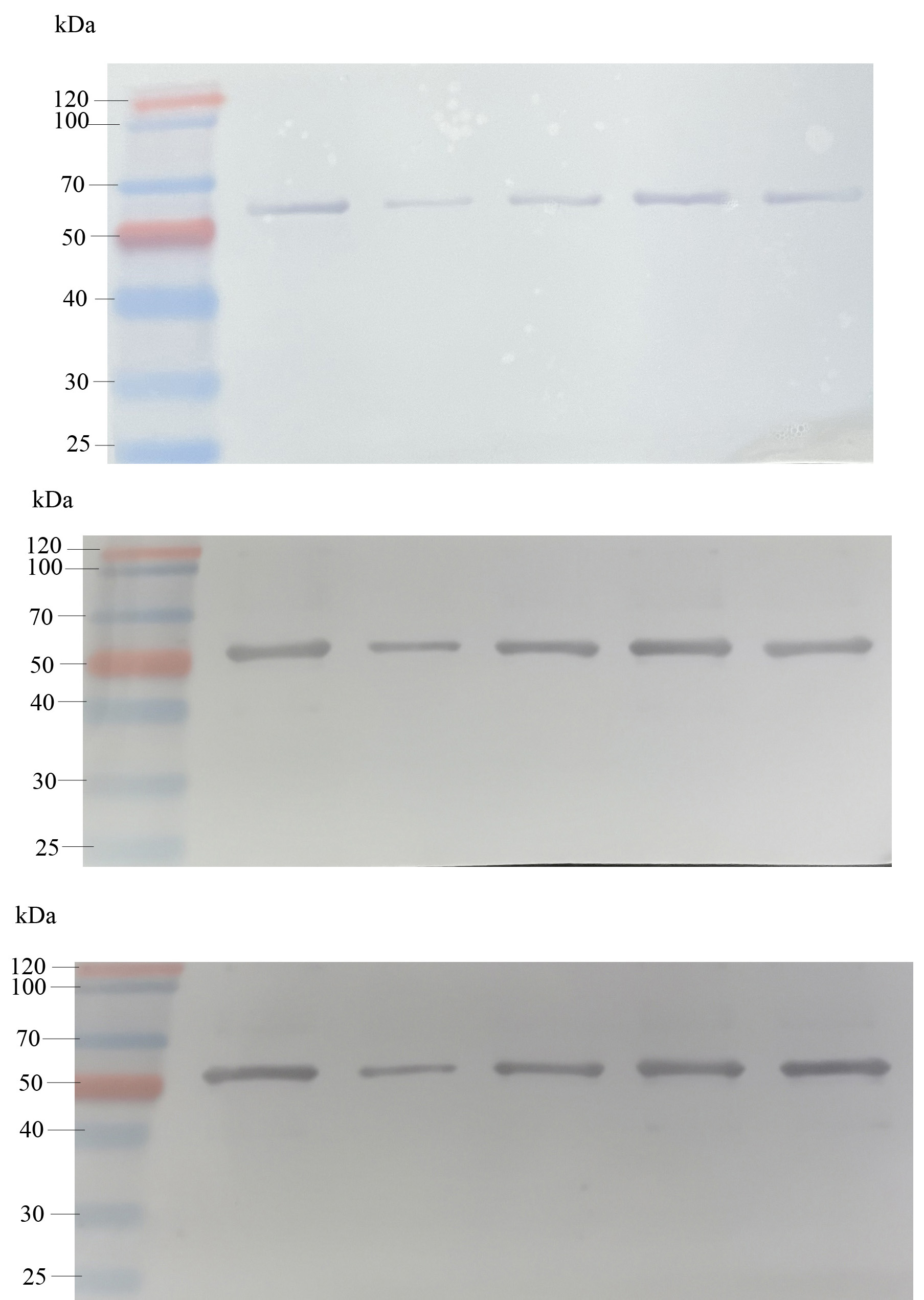

Supplement: Supplementary file 3 [file Image_3.JPEG]
